# Acute Cholecystitis Complicated with Gallbladder Perforation and Peritonitis Caused by Vancomycin-resistant Lactobacillus paracasei

**DOI:** 10.7759/cureus.7476

**Published:** 2020-03-30

**Authors:** Christina Lee, Nicha Wongjarupong, Muthu Narayan, Anne C Melzer

**Affiliations:** 1 Internal Medicine, University of Minnesota Medical School, St. Paul, USA; 2 Internal Medicine, University of Minnesota School of Medicine, Minneapolis, USA; 3 Infectious Diseases, Minneapolis Veterans Affairs Health Care System, Minneapolis, USA; 4 Pulmonary, Critical Care, Allergy and Sleep, Minneapolis Veterans Affairs Health Care System, Minneapolis, USA

**Keywords:** gallbladder perforation, peritonitis, vancomycin-resistant lactobacillus, acute cholecystitis

## Abstract

A 66-year-old female has a medical history of remote subarachnoid hemorrhage and dysphagia. She presented with acute onset of right upper quadrant abdominal pain. Ultrasound showed acute cholecystitis, and subsequent CT scan of the abdomen and pelvis showed gallbladder perforation. The patient’s hospital course was complicated with peritonitis, and bile culture grew vancomycin-resistant *Lactobacillus paracasei.* This case report will focus on an unusual case, in which *Lactobacillus* acts as the primary pathogen in peritonitis secondary to an cholecystitis-induced gallbladder perforation. There are four other case reports worldwide that illustrate *Lactobacillus* species as the primary pathogen in cholecystitis, only one of which was complicated with peritonitis.

## Introduction

*Lactobacillus* species are gram-positive, facultative anaerobic or microaerophilic, lactic-producing bacilli, which are commensal bacteria that normally reside in the mouth flora, gastrointestinal tract, and the female genital tract. *Lactobacillus* spp. have a mutualistic relationship with the human body and have been shown to play a key role in preventing colonization and adhesion by potential pathogens in the colon and are thus known as probiotics [1]. Additionally, the use of probiotics is also shown to enhance innate immunity and has a role in certain diseases such as inflammatory bowel disease [2,3]. However, *Lactobacillus* ssp. are also known to be pathogenic, causing endocarditis, bacteremia, peritonitis, and meningitis [4].

We report an unusual case of a 66-year-old female who was diagnosed with acute cholecystitis complicated by gallbladder perforation and peritonitis, from which *Lactobacillus paracasei* was isolated as the only pathogen. 

## Case presentation

A 66-year-old female presented with an acute onset of right upper quadrant abdominal pain and near syncope. Her medical history was notable for remote subarachnoid hemorrhage and aneurysm, with sequelae including incomplete quadriparesis and hydrocephalus requiring ventriculoperitoneal (VP) shunt, extensive bifrontal encephalomalacia, and dysphagia. Due to her history of brain injury, she was unable to give further details about her new onset abdominal pain.

On initial physical examination, vital signs were blood pressure 100/65 mmHg and heart rate 130 beats/min, and she was afebrile. There was no scleral icterus. Her abdomen was distended, exhibiting diffuse tenderness throughout her abdomen, worse in the right upper quadrant with voluntary guarding.

**Figure 1:**
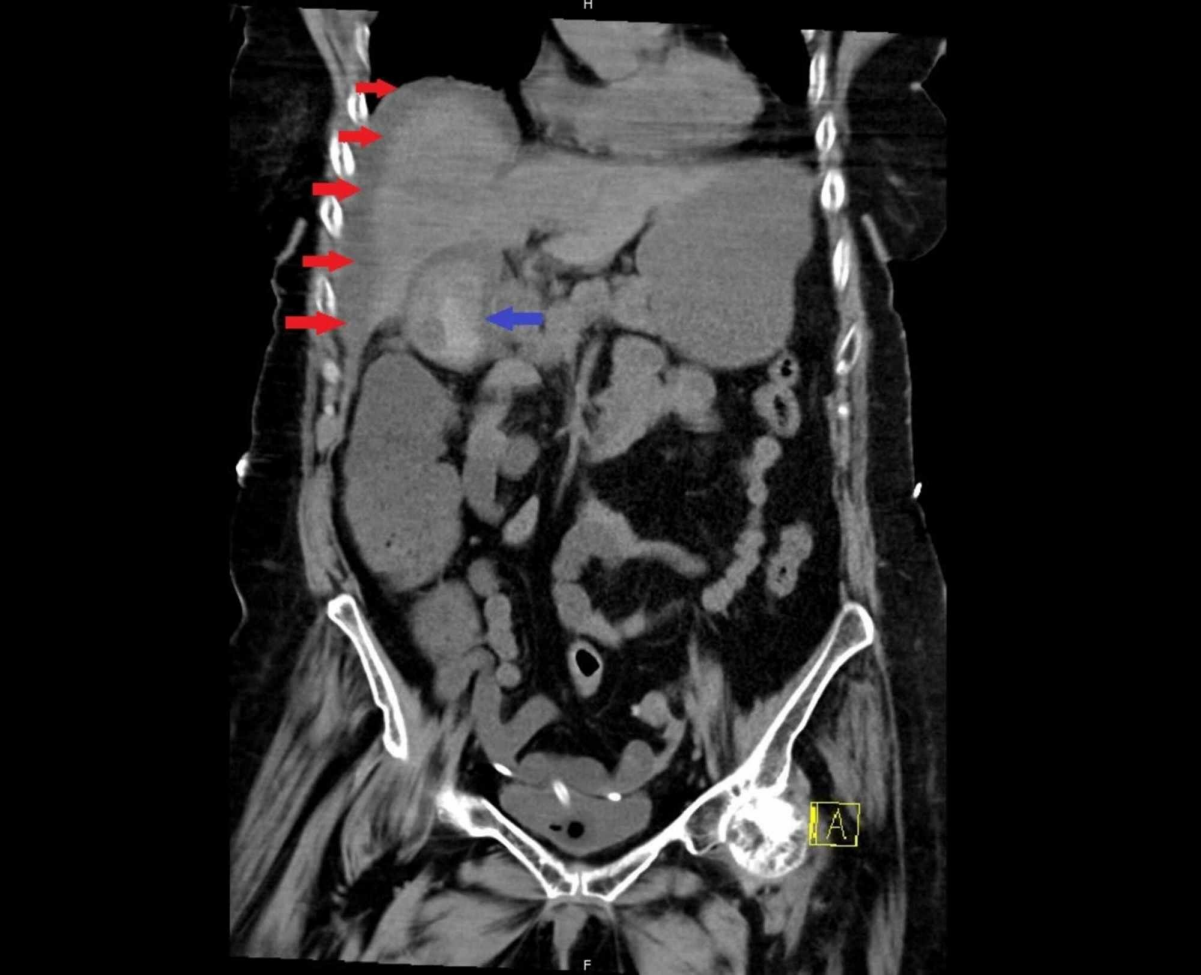
CT scan of the abdomen and pelvis showing perihepatic fluid (red arrows) secondary to acute cholecystitis complicated by gallbladder perforation (blue arrow).

Laboratory test results were significant for elevated white blood cell 18 K/cmm, lactate 8 mmoL/L, and C-reactive protein 58 mg/L. Her liver function labs, including her total bilirubin, were all within normal limits. Abdominal ultrasonography suggested acute cholecystitis with a positive sonographic Murphy’s sign. A subsequent CT scan of the abdomen and pelvis showed cholelithiasis with gallbladder wall enhancement and thickening, with pericholecystic fluid, also consistent with acute cholecystitis; the CT scan also showed lateral and inferior perihepatic fluids, due to gallbladder perforation (Figure 1).

Given her clinical decompensation with severe sepsis upon arrival to the medical intensive care unit (MICU) that evening, she was immediately and empirically started on both vancomycin and piperacillin/tazobactam, even prior to general surgery examining the patient. Ultimately, general surgery deemed her a poor surgical candidate, given her comorbidities, probable gallbladder perforation complicated by peritonitis, and her clinical decompensation with severe sepsis

**Figure 2:**
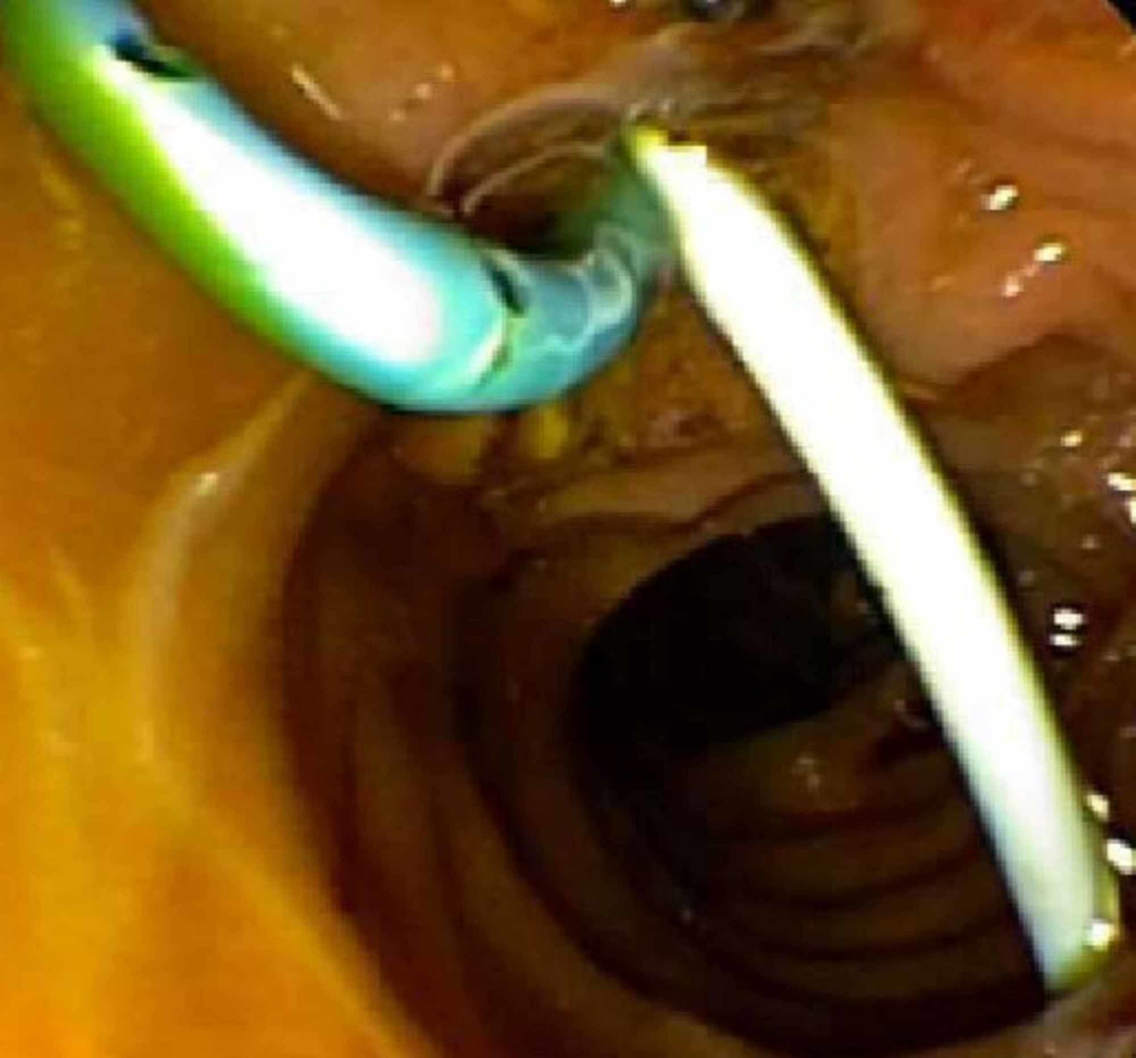
Tails of the two stents placed during an endoscopic retrograde cholangiopancreatography can be seen in the duodenum. One stent is placed in the common bile duct and the other stent is placed in the cystic duct.

Interventional radiology was consulted for an intra-abdominal drain placement, but due to a decompressed, perforated gallbladder, only a perihepatic drain was able to be placed on the second day of arrival to the MICU. Bile fluid from the perihepatic drain was obtained for cultures approximately one day after initiating antibiotics. The drain produced turbid fluid with gram-positive rods on stain. Although her cerebrospinal fluid did not show any bacterial infection, her VP shunt was externalized since it coursed through the site of infection. With the perihepatic drain placement, she clinically improved with regard to her mentation, abdominal pain, and leukocytosis; however, she continued to have high-output drainage in excess of 700 cc/daily of perihepatic fluid, initially turbid and subsequently clear and bilious. To redirect the high-output flow of bile, gastroenterology performed an endoscopic retrograde cholangiopancreatography-guided biliary sphincterotomy and stent placement, retrieving five gallstones from the gallbladder, and placing two stents, one in the common bile duct and one in the cystic duct (Figure 2). Perihepatic drain output dramatically declined thereafter, and the patient continued to improve clinically.

On day 7 of her hospital stay in the ICU, her bile fluid culture, which was collected from the perihepatic drain approximately one day after initiating antibiotics, grew only *Lactobacillus paracasei,* which was resistant to vancomycin, cefotaxime, and metronidazole, but sensitive to piperacillin/tazobactam. Vancomycin was discontinued after a total of seven days. A repeat CT scan of abdomen showed diminished perihepatic fluid and signs of improved inflammation, and piperacillin/tazobactam was stopped after a total of 11 days.

Overall, the patient continued to improve each day with the perihepatic drain in place, and while on piperacillin/tazobactam.

Prior to transferring out of the ICU, the patient had her perihepatic drain successfully removed. Fortunately, her VP shunt remained sterile throughout this process and was eventually removed due to the patient demonstrating minimal output of cerebrospinal fluid and showing no signs of hydrocephalus on CT head. She spent a total of four weeks in the ICU and was transferred to the medicine floor without recurrence of her symptoms and with her mentation back to her baseline. Notably, the patient and her family had previously been recommended to have cholecystectomy but had declined.

## Discussion

The critical discussion is whether or not the *Lactobacillus* spp., which were cultured from the perihepatic drain of our patient, were a contaminant or the primary pathogenic organism of this case. As mentioned earlier in the above section, cultures from the perihepatic drain were sent after the initiation of antibiotics, because antibiotics were started immediately upon arrival to the MICU, given the patient’s severe sepsis. The MICU team made an executive decision to immediately and empirically start antibiotics, because of her significant clinical decompensation. Although both consultant teams, including general surgery and interventional radiology, were timely in their response, the MICU team deemed it necessarily to start antibiotics immediately to stabilize the patient, while waiting for our consultants’ expertise. Although bile fluid cultures from the perihepatic drain were collected one day after initiation of vancomycin and piperacillin/tazobactam, we do not believe that this was sufficient time to eliminate other potential organisms. Thus, we believe that the* Lactobacillus* spp. isolated from our patient was the primary pathogen as opposed to a mere contaminant, especially because this antibiotic-resistant strain of* Lactobacillus *was isolated from a normally sterile site in very high concentrations. *Lactobacillus* is usually isolated as part of a polymicrobial infection, but for our patient,* Lactobacillus* was isolated alone, which further supports that* Lactobacillus *was acting as the primary pathogen in this patient’s case of acute cholecystitis.

Additionally, there are some differences in pathogenicity among the *Lactobacillus* spp.* Lactobacillus paracasei*, which was isolated from our patient’s drain, along with *Lactobacillus rhamnosus* is mostly known to be associated with infections, whereas *Lactobacillus gasseri *can be both pathogenic and a colonizer, further supporting *Lactobacillus paracasei *as the primary pathogen in our patient’s case [5].

In a retrospective study of more than 200 *Lactobacillus*-related cases, there were no reports of* Lactobacillus* as the primary pathogen in the setting of cholecystitis, making our patient case unique [4]. Infection of the biliary tract caused by *Lactobacillus *spp. is rare; bile fluid is typically infected with* Escherichia coli, Enterococcus* spp.,* Klebsiella* spp., *Enterobacter* spp., *Clostridium* spp., and *Bacteroides fragilis* group [6]. To the best of our knowledge, there are only four other case reports worldwide thus far reporting primary pathogenic *Lactobacillus* in the setting of acute cholecystitis, only two of which are from within the United States and only one of which was complicated with peritonitis just like our patient’s case [7-10].

Upon further discussion with the patient’s spouse, he reported that the patient ate three to four containers (32 oz each per container) of yogurt per day, as meal substitutes due to her history of dysphagia. Although *Lactobacillus* is known to be the most common probiotic found in foods like yogurt, there are no studies to support a direct association between yogurt/probiotic consumption and infection. For instance, a large cohort study in Stockholm, which looked at the incidence of *Lactobacilli*-induced bacteremia in 59 bacteremia cases, identified zero cases of bacteremia caused by *Lactobacillus (Lactobacillus paracasei, Lactobacillus acidophilus, Lactobacillus rhamnosus)* [11]. In all the 59 bacteremia cases, none of the isolates were identical to the probiotic strains by randomly amplified polymorphic DNA polymerase chain reaction method [11]. Until proven otherwise by well-developed cohort studies in the future, we do not believe that there was a correlation between our patient’s yogurt consumption and the* Lactobacillus* infection, and there could have been a recall bias.

## Conclusions

Although* Lactobacillus* spp. are rarely detected in blood cultures and are deemed to have low pathogenicity in literature, there may be a need for heightened consideration for *Lactobacillus* spp. as a possible primary pathogen in patients presenting with signs and symptoms of acute cholecystitis. This heightened suspicion can lead to more appropriate antibiotic selection, with the knowledge of potential resistance of *Lactobacillus* to vancomycin. We believe that this knowledge about vancomycin-resistant *Lactobacillus* spp. will aid in better management with proper antibiotic coverage and thus, lead to better outcomes by preventing the possible sequelae of unfortunate events from the complications of an acute cholecystitis.
